# Bone morphogenetic protein 12: a novel target for cardiovascular disease

**DOI:** 10.1186/s43556-026-00609-y

**Published:** 2026-10-29

**Authors:** Stephania C. Isaza, Miguel Á. Hernández-García, Carlos Ernesto Fernández-García, Carolina Pérez-Utrera, María Álvarez-Rubal, José M. Muñoz, Alfonsa Friera, Carmelo García-Monzón, Almudena Gómez-Hernández, Pedro Landete, Javier Egea, Fernando Alfonso, Águeda González-Rodríguez

**Affiliations:** 1 Unidad de Investigación, Hospital Universitario Santa Cristina, Instituto de Investigación Sanitaria-Princesa IIS-IP, Madrid, Spain; 2 Instituto de Investigaciones Biomédicas Sols-Morreale (IIBM), Consejo Superior de Investigaciones Científicas-Universidad Autónoma de Madrid, Madrid, Spain; 3 Servicio de Radiodiagnóstico, Hospital Universitario de La Princesa, Instituto de Investigación Sanitaria-Princesa IIS-IP, Madrid, Spain; 4 Laboratorio de Enfermedades Hepáticas y Vasculares, Departamento de Bioquímica y Biología Molecular, Facultad de Farmacia, Universidad Complutense de Madrid, Madrid, Spain; 5 Servicio de Neumología, Hospital Universitario de La Princesa, Instituto de Investigación Sanitaria-Princesa IIS-IP, Madrid, Spain; 6 Universidad Autónoma de Madrid, Madrid, Spain; 7 Servicio de Cardiología, Hospital Universitario de La Princesa, Instituto de Investigación Sanitaria-Princesa IIS-IP, Madrid, Spain; 8 Centro de Investigación Biomédica en Red de Enfermedades Cardiovasculares (CIBERCV), Madrid, Spain; 9 Centro de Investigación Biomédica en Red de Diabetes y Enfermedades Metabólicas Asociadas (CIBERDEM), Madrid, Spain

**Keywords:** Bone morphogenetic protein, BMP12, Vascular calcification, Pathological wall remodeling, Cardiovascular disease, Atherosclerosis

## Abstract

Bone morphogenetic protein 12 (BMP12) is known to regulate mesenchymal differentiation and extracellular matrix remodeling. Given its structural and signaling similarities to BMP2, which promotes osteogenic phenotypic switching and pro-atherogenic responses in vascular smooth muscle cells (VSMCs), BMP12 may also contribute to vascular remodeling and atherosclerosis progression. This translational study aimed to unravel BMP12 involvement in cardiovascular disease (CVD). BMP12 effects on atherogenesis-related processes in VSMCs in absence or presence of BMP type I receptor inhibitors (DMH2 and LDN-193189) were evaluated. Additionally, circulating BMP12 levels were measured in a well-characterized cohort of patients with CV risk factors and correlated with cardiometabolic disorders and vascular damage markers. Results from the experimental study showed increased migration and calcification in BMP12-treated VSMCs. Mechanistically, BMP12 induced expression of osteogenic markers (RUNX2 and ALP) and genes related to cellular migration (*Mmp9*), while reducing contractile marker *Desmin*. These pro-atherogenic effects were blocked using BMP type I receptor inhibitors. Furthermore, the clinical study revealed higher serum BMP12 levels in patients with CVD and type 2 diabetes. Moreover, circulating BMP12 concentrations were elevated in patients with carotid and femoral plaques, correlating with plaque volume. Notably, BMP12 emerged as an independent predictor of femoral atherosclerosis. Collectively, these findings identify BMP12 as a previously unrecognized mediator of vascular remodeling, promoting pro-atherogenic effects in VSMCs through canonical BMP signaling. The association between elevated circulating BMP12 levels and subclinical vascular damage further supports its potential value as a contributor to atherosclerosis progression and as a promising novel candidate biomarker for CV risk.

**Supplementary Information:**

The online version contains supplementary material available at https://doi.org/10.1186/s43556-026-00609-y.

## Introduction

Cardiovascular (CV) disease (CVD) is one of the leading causes of mortality worldwide [1]. Although CVD-related mortality has been reduced in recent years due to new prevention strategies and the development of pharmacological treatments, it continues to be responsible for a large number of deaths, which are, in most cases, secondary to atherothrombosis [2]. Atherosclerosis is highly prevalent and forms the underlying pathophysiology of coronary artery disease (CAD) [3], peripheral artery disease (PAD) [4], and cerebrovascular disease, which is characterized by carotid atherosclerosis [5]. These pathologies share a common pathogenesis and key risk factors for their development, including smoking, dyslipidemia, hypertension, and type 2 diabetes (T2D) [6]. Among these manifestations, PAD presents a clinical challenge: plaque rupture, erosion, or progressive luminal narrowing in the lower extremities can elicit superimposed atherothrombosis and subsequent vessel occlusion, leading to critical limb ischemia, a high risk of amputation, and an elevated incidence of systemic CV death [7]. Despite its severity and high prevalence, atherothrombotic disease is still often underdiagnosed and insufficiently treated, resulting in a growing medical and economic burden on healthcare systems driven by chronic disability and the considerable costs associated with vascular revascularization procedures [8, 9]. Additionally, the growing incidence of atherosclerosis in low- and middle-income nations represents a major and rising global public health challenge [10].

Currently, there is no known biochemical marker of atherothrombosis with sufficient specificity and sensitivity to be used in clinical practice. Therefore, surrogate markers, such as intima-media thickness (IMT) or the ankle-brachial index (ABI), are widely used for atherosclerosis screening [11]. Additionally, emerging surrogates of visceral adiposity, such as the Lipid Accumulation Product (LAP), an index of central lipid overaccumulation, and the Visceral Adiposity Index (VAI), a sex-specific indicator of adipose tissue distribution, have emerged as potent predictors of CV risk [12, 13]. Ultimately, the identification of novel biomarkers could facilitate the diagnosis of atherosclerosis, improve our understanding of its onset and prognosis, and aid in assessing patients' response to treatment.

Bone morphogenetic proteins (BMPs) are secreted glycoproteins that were first described as osteoinductive soluble factors and belong to the transforming growth factor β (TGF-β) superfamily [14]. Regarding CVD, studies indicate that BMP activation frequently results in vascular inflammation, triggering calcification of vascular smooth muscle cells (VSMCs) and endothelial dysfunction [15]. Far from being a passive mineral deposition, vascular calcification is a highly regulated biological process driven by the phenotypic switching of VSMCs. In response to pro-atherogenic stimuli, VSMCs lose their contractile markers and transdifferentiate into osteoblast-like cells. This mineral remodeling not only increases arterial stiffness but also dictates plaque vulnerability and clinical outcomes, making the identification of the molecular signals that trigger this osteogenic transition a priority in CV research [16]. Upregulated BMP2, BMP4, and BMP6 levels have been found in atherosclerotic lesions, and are associated with pro-atherogenic effects such as endothelial dysfunction and osteogenic differentiation [17, 18]. Canonical BMP signaling occurs through heterotetrameric type I (ALK2, ALK3, and ALK6) and type II receptors that phosphorylate SMAD1/5/9, which form a complex with SMAD4, regulating the expression of many downstream genes [19]. In terms of vascular biology, type I BMP receptor inhibition reduces vascular calcification and atherosclerosis in different experimental models [20, 21]. Indeed, pharmacological inhibition of these type I receptors using LDN-193189 effectively disrupts BMP2-induced osteogenic transdifferentiation of VSMCs [22]. In these cells, BMP2 enhances cellular migration [23] and stimulates vascular calcification by prompting the induction of the osteogenic phenotype [24]. In this context, significant associations between BMP2 expression and the presence of unstable plaques have been described [25], and plasma BMP2 levels positively correlate with the calcium density of plaques in hyperglycemia-induced vascular calcification [26].

Despite the well-established role of several BMP family members in vascular calcification, the potential involvement of BMP12 (also known as growth differentiation factor 7, GDF7) in CVD remains largely unexplored. Given its recognized functions in mesenchymal differentiation and extracellular matrix regulation [27, 28], BMP12 may participate in vascular remodeling processes associated with atherosclerosis progression. This hypothesis is further supported by the marked structural and signaling similarities between BMP12 and BMP2, which share substantial sequence homology and conserved canonical BMP signaling through SMAD1/5/9 activation [29]. Given the established role of BMP2 in promoting the osteogenic phenotypic switching of VSMCs, BMP12 may similarly contribute to vascular remodeling through the activation of the canonical BMP signaling pathway.

Through a translational research approach, the experimental study herein demonstrated that BMP12 induced both migration and calcification in VSMCs, two key processes in the pathological remodeling of the arterial wall and the development of vascular stiffness. These pro-atherogenic effects were mediated by the canonical BMP signaling pathway. Furthermore, the present clinical study revealed elevated serum levels of BMP12 in patients with CVD and T2D, positioning this BMP protein as an independent predictor of femoral atherosclerosis.

## Results

### BMP12 promotes osteogenic phenotypic switching and pro-atherogenic responses in VSMCs

We first analyzed *BMP12* mRNA levels in atherosclerotic lesions using independent datasets from the NCBI Gene Expression Omnibus (GEO) database. Similar to *BMP2*, this transcriptomic analysis confirmed consistent expression of *BMP12* across different human vascular territories affected by atherosclerosis (Fig. S1). These findings support the involvement of BMP12 in the atherosclerotic microenvironment and suggest a potential contribution to vascular remodeling.

**Fig. 1:**
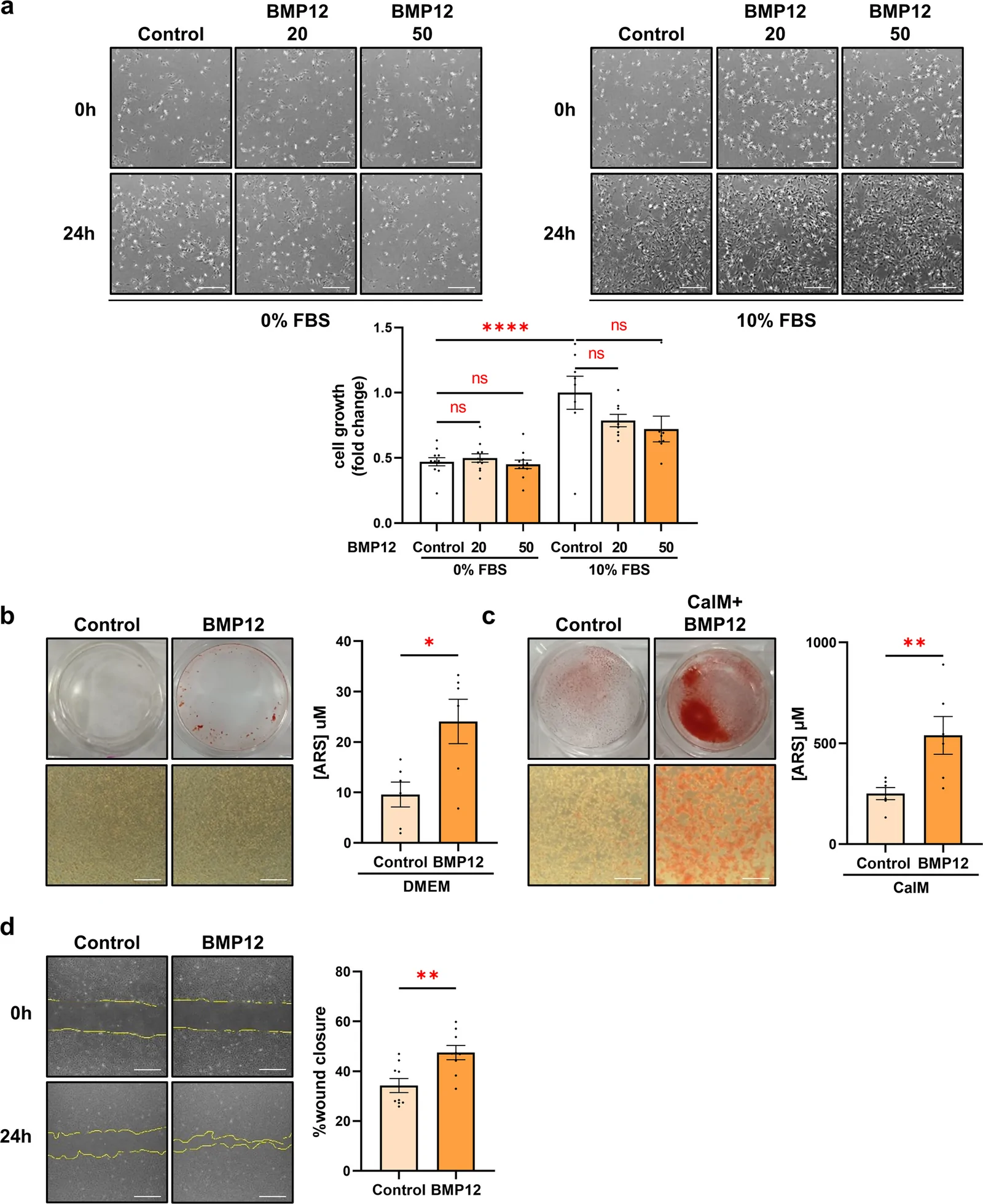
BMP12 enhances calcification and migration of primary mouse VSMCs. **a** Representative phase-contrast images (4X, scale bar 500 µm) and quantification of crystal violet staining. Data are expressed as fold change relative to control condition 10% FBS at 24 h (Control 10% FBS, 1), presented as mean ± SEM and compared using one-way ANOVA with Tukey’s post hoc test. Experimental conditions: Cells were treated with recombinant mouse BMP12 at 20 or 50 ng/mL in the absence (*n* = 11 for each condition) or presence (*n* = 8 for each condition) of serum at 0 and 24 h. **b**, **c** Representative images and phase-contrast images (4X, scale bar 500 µm) of Alizarin Red staining (ARS) and their corresponding quantification. Data are expressed as ARS concentration (µM), presented as mean ± SEM and compared using unpaired t-test. Experimental conditions: Cells were cultured in the absence or presence of recombinant BMP12 (20 ng/mL) either in medium with normal (DMEM, Control) (**b**) or elevated phosphate concentration (calcifying medium, CalM) (**c**) for 10 days (*n* = 6 for each condition). **d** Representative phase-contrast images of the wound-healing assay (4X, scale bar 500 µm) and its quantification. Data are expressed as percentage of wound closure (%), presented as mean ± SEM and compared using unpaired t-test. Experimental conditions: Cells were cultured in the absence or presence (*n* = 9 for each condition) of recombinant BMP12 (20 ng/mL) in DMEM 0% FBS. *p < 0.05, **p < 0.01, and ****p < 0.001

On this basis, the present experimental study focused on evaluating the effect of BMP12 on VSMC biology. To this end, we characterized different key processes related to atherogenesis, such as proliferation, migration, and calcification in murine primary VSMCs in the presence or absence of recombinant BMP12. First, the effect of this BMP protein on VSMC proliferation was explored. Although increased proliferation was observed in cells cultured in the presence of serum, BMP12 treatment did not influence the proliferative capacity of these cells (Fig. 1a).

To test whether recombinant BMP12 treatment affects VSMC extracellular matrix calcification, primary VSMCs were cultured in a medium with elevated inorganic phosphate (Pi) concentration (calcifying medium, CalM). After staining with Alizarin Red S, it was observed that cells treated with BMP exhibited significantly more calcium deposits (Fig. 1b), and this BMP12-induced calcification was more pronounced in the presence of calcifying medium (Fig. 1c).

To assess VSMC migration, wound healing assays were performed. BMP12 protein promoted wound closure and migratory behavior in VSMCs, as the average wound closure in untreated cells (controls) was approximately 35%, while it reached 50% in BMP12-treated cells (Fig. 1d).

**Fig. 2:**
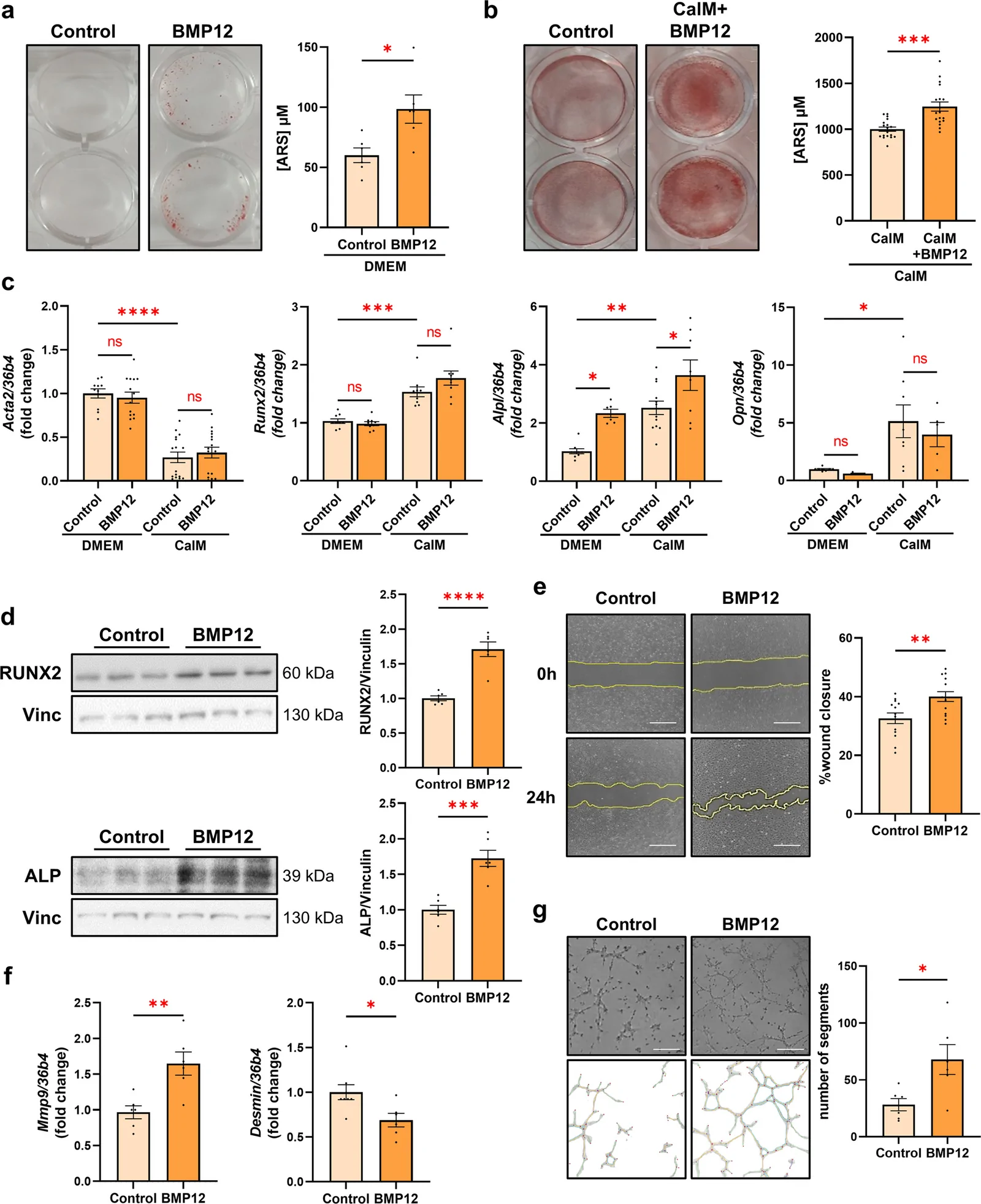
BMP12 promotes calcification and migration in a murine VSMC cell line. **a**, **b** Representative images of Alizarin Red staining (ARS) and its quantification. Data are expressed as ARS concentration (µM), presented as mean ± SEM and compared using unpaired t-test. Experimental conditions: Cells were cultured in the absence or presence of recombinant BMP12 (20 ng/mL) in either medium with normal (DMEM, Control) (n = 6 for each condition) (**a**) or elevated phosphate concentration (calcifying medium, CalM) (n = 18 for each condition) (**b**) for 3 days. **c** mRNA levels of *Acta2*, *Runx2*, *Alpl* and *Opn* determined by RT-qPCR and normalized to *36b4* gene expression. Data are expressed as fold change relative to control condition, presented as mean ± SEM and compared using one-way ANOVA with Tukey’s post hoc test. Experimental conditions: Cells were treated with or without recombinant BMP12 (20 ng/mL) in DMEM or CalM for 8 h for *Runx2* (n = 9–12 for each condition), 24 h for *Acta2* (n = 14–16 for each condition) and 48 h for *Alpl* (n = 6–8 for each condition) *and Opn* (n = 5–8 for each condition). **d** Representative blots with the indicated antibodies and the corresponding quantification. Data are expressed as mean ± SEM, presented relative to the control condition, and compared using unpaired t-test. Experimental conditions: Cells were treated with or without recombinant BMP12 (20 ng/mL) for 16 h in CalM for RUNX2 and for 24 h in DMEM for ALP (n = 6 for each condition). **e** Representative phase-contrast images of the wound-healing assay (4X, scale bar 500 µm) and its quantification. Data are expressed as percentage of wound closure (%), presented as mean ± SEM and compared using unpaired t-test. **f** mRNA levels of *Mmp9* and *Desmin* determined by RT-qPCR and normalized to *36b4* gene expression. Data is expressed as fold change relative to control condition, presented as mean ± SEM and compared using unpaired t-test. **g** Representative phase-contrast images (4X, scale bar 500 µm), analyzed images of the Matrigel migration assay and its quantification. Data are expressed as number of segments, presented as mean ± SEM and compared using unpaired t-test. Experimental conditions: Cells were treated with or without recombinant BMP12 (20 ng/mL) in DMEM 0% FBS for 24 h for wound-healing assay (n = 13–14 for each condition), 1 h for *Mmp9* (n = 6 for each condition), 24 h for *Desmin* (n = 6–8 for each condition) and 6 h for Matrigel migration assay (n = 6 for each condition). *p < 0.05, **p < 0.01, ***p < 0.005 and ****p < 0.001

To further investigate the molecular mechanisms underlying the effects of BMP12 on vascular biology, we performed a series of experiments using a murine VSMC cell line. Consistent with the results obtained in primary cells, the presence of this protein did not alter the proliferative capacity of VSMCs regardless of the BMP12 dose or exposure time (Fig. S2). Next, calcification analysis indicated that BMP12 increased VSMC matrix mineralization in the presence of normal Pi conditions (Fig. 2a) and in calcifying medium (Fig. 2b) compared to untreated cells. To better understand the osteogenic effect induced by BMP12, we measured the mRNA levels of *Acta2* (the gene encoding α-smooth muscle actin, αSMA), and *Runx2*, *Alpl* and *Opn* (genes encoding runt-related transcription factor 2, RUNX2; alkaline phosphatase, ALP; and osteopontin, OPN, respectively) in VSMCs. Whereas no differences were observed in the modulation of *Acta2* and *Opn* expression, BMP12 treatment induced the upregulation of *Runx2* and *Alpl* expression alongside their respective protein levels, confirming the osteogenic effect of this BMP protein on VSMCs (Fig. 2c, d).

Moreover, the wound healing assay demonstrated that BMP12 also influenced the migratory ability of the VSMC cell line (Fig. 2e). At the molecular level, BMP treatment significantly increased mRNA levels of *Mmp9* (the gene encoding matrix metalloproteinase 9, MMP9), which is important for VSMC migration [30], whereas mRNA expression of *Desmin* was significantly reduced (Fig. 2f). Cellular migration was also assessed by Matrigel-based segment formation assay. As shown in Fig. 2g, BMP12 increased segment/network formation of VSMCs on Matrigel compared with untreated cells, suggesting enhanced extracellular matrix-dependent remodeling behavior.

### Inhibition of canonical BMP signaling suppresses BMP12-induced osteogenic effects in VSMCs

In order to further define the contribution of BMP12 signaling to vascular remodeling, we investigated the effects of pharmacological inhibition of canonical BMP signaling using selective small-molecule inhibitors targeting type I receptor serine/threonine kinase activity.

**Fig. 3:**
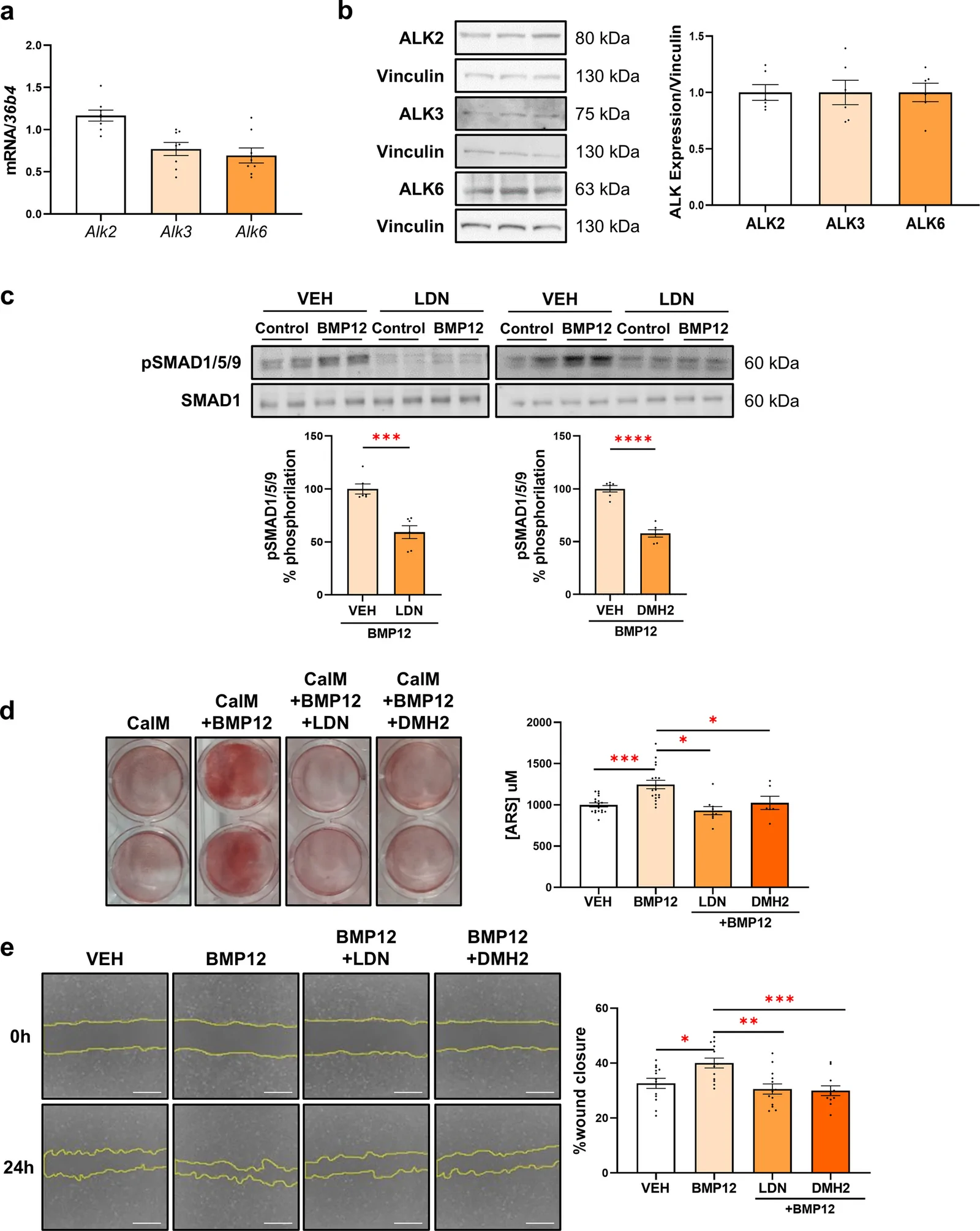
Inhibition of type I BMP receptor family suppresses calcification and migration induced by BMP12 in a murine VSMC cell line. **a** *Alk2, Alk3* and *Alk6* determined by RT-qPCR and normalized to *36b4* gene expression. Data are presented as mean ± SEM (*n* = 8 for each condition). **b** Representative blots with the indicated antibodies and their corresponding quantification. Data are presented as mean ± SEM (*n* = 6 for each condition). Experimental conditions: Cells were cultured in DMEM 0% FBS for 24 h. **c** Representative blots with the indicated antibodies and the corresponding quantification. Data are expressed as percentage relative to the control group, presented as mean ± SEM and compared using unpaired t-test. Experimental conditions: Cells were pre-treated with the inhibitors LDN-193189 (LDN, 300 nM), DMH2 (400 nM) or vehicle (VEH) for 1 h and further stimulated with or without recombinant BMP12 (20 ng/mL) for 8 h (n = 6 for each condition). **d** Representative Alizarin Red staining (ARS) and corresponding quantification. Data are expressed as concentration of ARS (µM), presented as mean ± SEM and compared using one-way ANOVA with Tukey’s post hoc test. Experimental conditions: Cells were pre-treated with LDN-193189 (LDN, n = 9) or DMH2 (n = 6) for 1 h prior to the stimulation with or without recombinant BMP12 (20 ng/mL) for 3 days in presence of calcifying medium (CalM) (n = 18 for Control and BMP12 conditions). **e** Representative phase-contrast images of wound-healing assay (4X, scale bar 500 µm) and its quantification. Data are expressed as percentage of wound closure (%), presented as mean ± SEM and compared using one-way ANOVA with Tukey’s post hoc test. Experimental conditions: Cells pre-treated with LDN-193189 (LDN, *n* = 13) or DMH2 (*n* = 11) for 1 h prior to the stimulation with or without recombinant BMP12 (20 ng/mL) or vehicle (VEH) for 24 h (n = 13 for Control and BMP12 conditions). *p < 0.05, **p < 0.01, ***p < 0.005 and ****p < 0.001

First, we confirmed the expression of type I BMP receptors (ALK2, ALK3, and ALK6) in the VSMC cell line (Fig. 3a, b). Next, VSMCs were pre-treated with the inhibitors and then stimulated with BMP12. Activation of SMAD1/5/9 was strongly induced upon BMP12 stimulation, whereas a reduction of BMP12-induced SMAD1/5/9 phosphorylation was observed using both DMH2 and LDN inhibitors (Fig. 3c).

As shown in Fig. 3d, BMP12 treatment further enhanced Pi-induced calcium accumulation; however, this effect was blocked by both inhibitors. Similarly, the increased migration observed upon BMP12 challenge was suppressed in the presence of DMH2 and LDN (Fig. 3e). Taken together, these findings indicate that the inhibition of canonical BMP signaling by targeting type I BMP receptors is sufficient to block BMP12-induced osteogenic effects in VSMCs.

### Clinical features of the study population

Our results suggest that BMP12 may trigger pathological vascular wall remodeling through the modulation of VSMC migration and contractility, processes that can lead to arterial stiffness and promote the onset of atherosclerosis. Based on these experimental findings, we next evaluated whether the effects of BMP12 on VSMCs are relevant to human disease by assessing circulating BMP12 levels in a cohort of 184 patients at CVD risk in order to assess its utility as a non-invasive biomarker of vascular pathology. A detailed description of the study cohort is provided in Table 1.

**Table 1:** Characteristics of the study population (*n* = 184)

Features	Study cohort (N = 184)
Age (years)	58.97 ± 8.75
Gender	
Women, n (%)	75 (40.8)
Men, n (%)	109 (59.2)
Body mass index (kg/m^2^)	30.17 ± 5.53
Arterial hypertension, n (%)	89 (48.4)
Current smoking, n (%)	47 (25.5)
Previous CVD, n (%)	25 (13.6)
Heart failure, n (%)	16 (8.7)
Atrial fibrillation, n (%)	13 (7.1)
Acute myocardial infarction, n (%)	10 (5.4)
Stroke, n (%)	7 (3.8)
Carotid atherosclerosis, n (%)	80 (43.5)
Plaques ≥ 0.20 mL, n (%)	10 (5.4)
Femoral atherosclerosis, n (%)	100 (54.3)
Plaques between 0.20 and 0.49 mL, n (%)	25 (13.6)
Plaques ≥ 0.50 mL, n (%)	10 (5.4)
Diabetes, n (%)	29 (15.8)
Glucose (mg/dL)	104.68 ± 27.48
Glycated Hemoglobin (%)	5.73 ± 0.70
HOMA-IR score	4.63 ± 6.68
Total cholesterol	193.48 ± 36.49
LDL cholesterol (mg/dL)	111.15 ± 33.20
HDL cholesterol (mg/dL)	54.74 ± 17.26
Triglycerides (mg/dL)	132.45 ± 77.51
ALT (IU/L)	23.33 ± 12.62
AST (IU/L)	22.25 ± 10.11
GGT (IU/L)	33.45 ± 26.68
Alkaline phosphatase (IU/L)	67.75 ± 19.01
Lactate dehydrogenase (U/L)	186.80 ± 38.88
C-reactive protein (mg/L)	0.40 ± 0.49
Albumin (g/dL)	4.40 ± 0.28
Platelets (10^9^/L)	228.20 ± 54.80
Serum Creatinine (mg/dL)	0.89 ± 0.18
Glomerular filtration rate (mL/min/1.73 m^2^)	84.50 ± 16.16

Data are presented as mean ± standard deviation or as number of cases (%). HOMA-IR, homeostatic model assessment of insulin resistance; HDL, high-density lipoprotein; LDL, low-density lipoprotein; ALT, alanine aminotransferase; AST, aspartate aminotransferase; GGT, gamma-glutamyltransferase

The mean age of the study cohort was 59 years, and 59.2% of the patients were men. Arterial hypertension was previously diagnosed in 48.4% and previous CVD in 13.6% of the patients. Carotid atherosclerotic plaques were detected in 43.5% and femoral atherosclerotic plaques in 54.3% of the patients. The mean body mass index (BMI) was slightly over 30 kg/m^2^, but the lipid profile, based on total, high-density lipoprotein (HDL), and low-density lipoprotein (LDL) cholesterol, and triglycerides, was within clinically acceptable values. A previous history of T2D was recorded in only 15.8% of the patients, although the cohort showed slightly elevated values of fasting glucose, glycated hemoglobin, and insulin resistance according to the homeostatic model assessment of insulin resistance (HOMA-IR). Taken together, these findings indicate that the cohort exhibited features of metabolic syndrome defined by the Adult Treatment Panel III (ATP III) criteria [31], making it a population with an increased risk of CVD and, therefore, suitable for CV risk studies.

### Serum BMP12 levels are elevated in patients with previous CVD and T2D

**Fig. 4:**
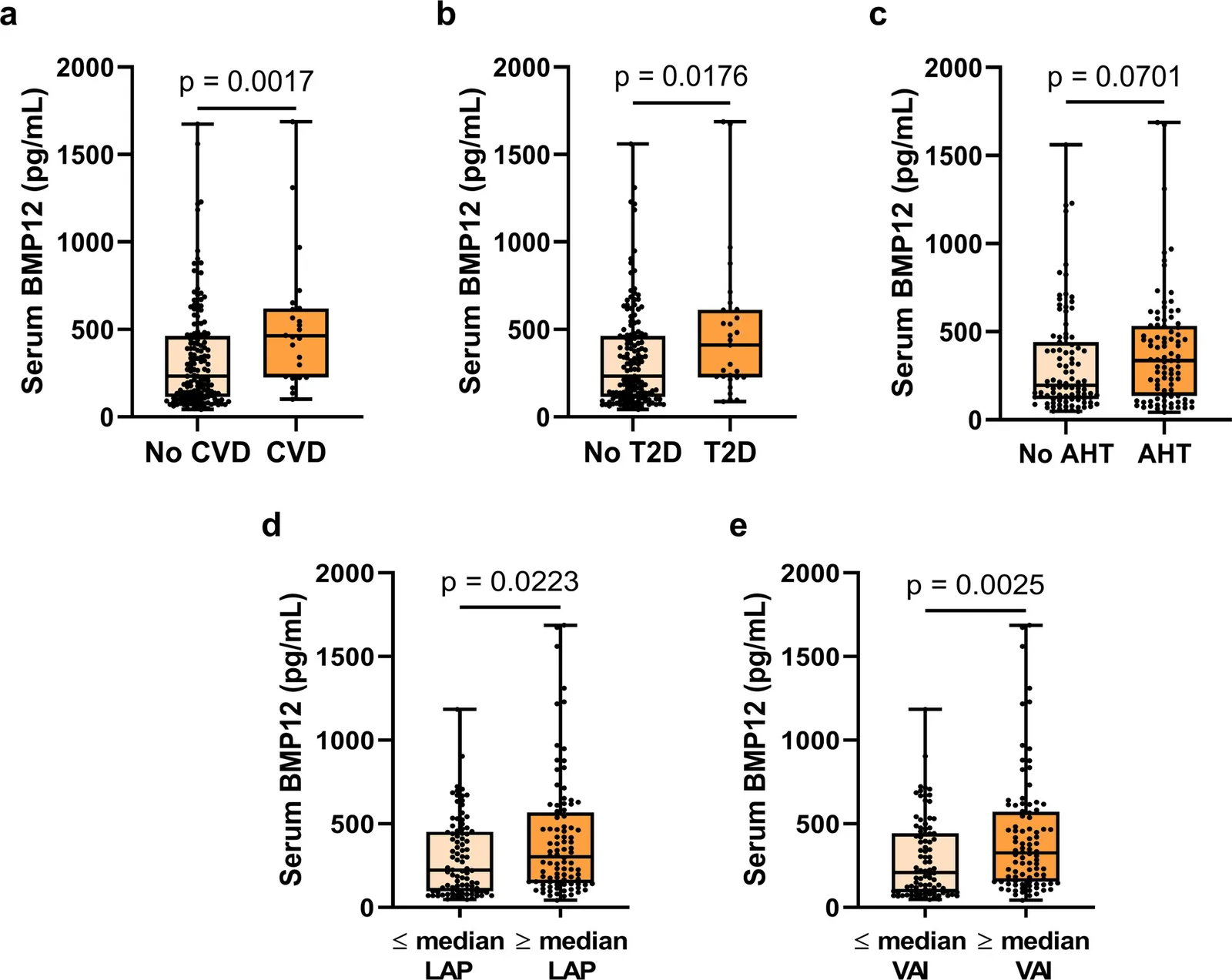
Serum BMP12 concentration is increased in patients with cardiovascular disease, type 2 diabetes, and with higher cardiovascular risk. **a**-**e** BMP12 concentration determined by ELISA in serum from the study population (n = 184) according to previous history of cardiovascular disease (CVD) (No CVD = 159, CVD = 24) (**a**), type 2 diabetes (T2D) (No T2D = 155, T2D = 29) (**b**), and arterial hypertension (AHT) (No AHT = 95, AHT = 89) (**c**), Lipid Accumulation Product (LAP, **d**) and Visceral Adiposity Index (VAI, **e**) scores (≤ median = 92, ≥ median = 92). Data are expressed as concentration of BMP12 (pg/mL), presented as mean ± SD and compared using the Mann–Whitney U test

First, serum BMP12 levels in our study cohort were analyzed according to the presence or absence of a previous history of CVD. Circulating levels of BMP12 were significantly higher in patients with a previous history of CVD, including heart failure, atrial fibrillation, acute myocardial infarction, and stroke (514.80 ± 363.93 vs. 329.83 ± 291.54 pg/mL, *p* = 0.002) (Fig. 4a), as well as in patients with T2D (482.83 ± 405.04 vs. 331.04 ± 281.36 pg/mL, *p* = 0.018) (Fig. 4b). Moreover, BMP12 levels were higher in patients with arterial hypertension (AHT), but these differences did not reach statistical significance (389.81 ± 317.15 vs. 322.31 ± 296.91 pg/mL, p = 0.070) (Fig. 4c). We also examined serum BMP12 concentrations in patients according to LAP and VAI levels, as these scores are associated with CV risk. In our study cohort, circulating BMP12 levels were significantly increased in the serum of patients with high LAP (298.80 ± 232.56 vs. 411.13 ± 360.79 pg/mL, *p* = 0.022) (Fig. 4d) or high VAI (289.24 ± 234.85 vs. 420.68 ± 356.01 pg/mL, p = 0.002) (Fig. 4e).

### Circulating BMP12 levels are increased in patients with carotid and femoral atherosclerosis

**Fig. 5:**
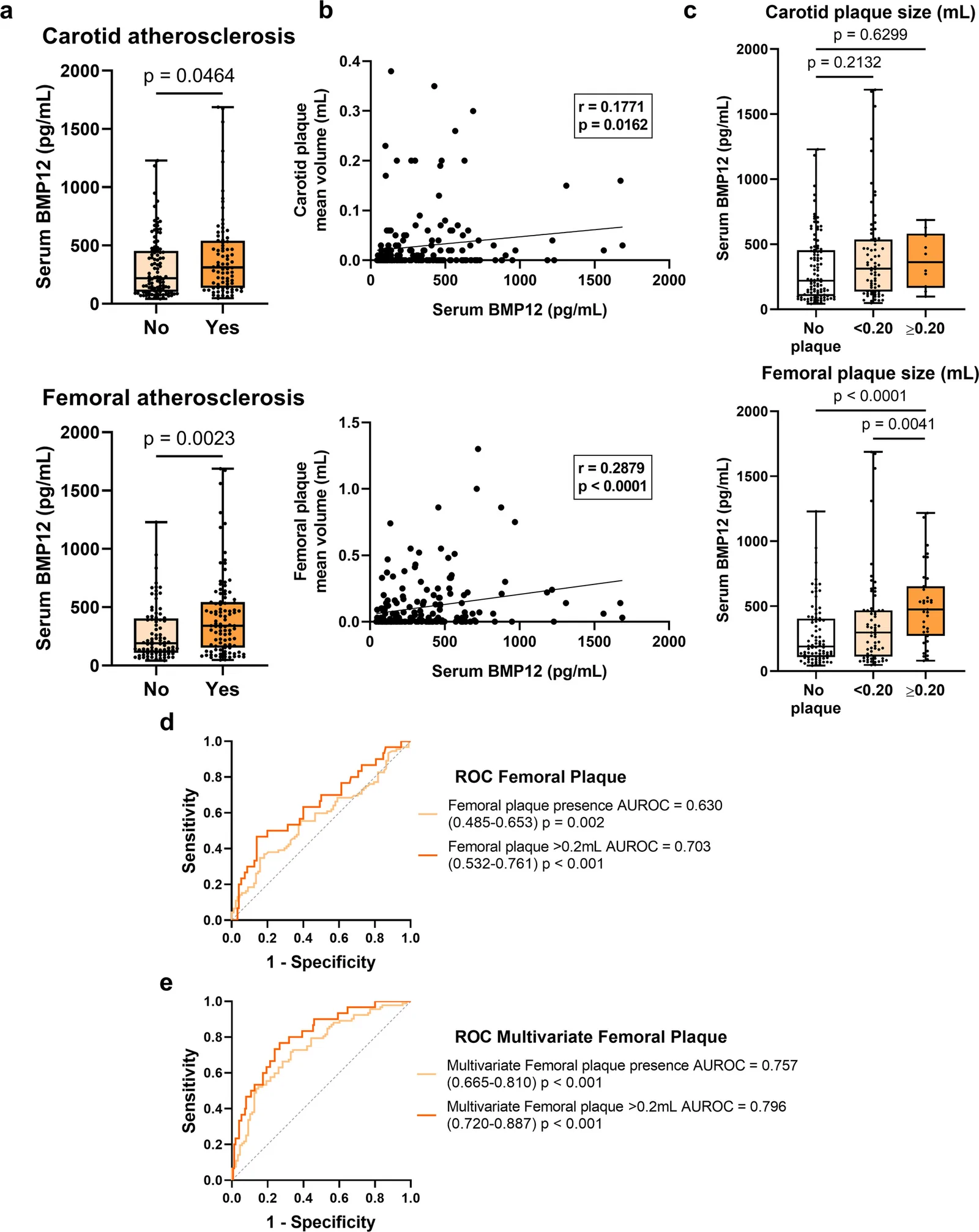
Serum BMP12 is elevated in patients that presented carotid and femoral atherosclerosis. **a** BMP12 concentration was determined by ELISA in serum from the study population (n = 184) according to the presence of carotid (upper panel; No = 104, Yes = 80) or femoral atherosclerosis (lower panel; No = 84, Yes = 100). Data are expressed as concentration of BMP12 (pg/mL), presented as mean ± SD and compared using Mann–Whitney U test. **b** Correlation of serum BMP12 concentration with carotid (upper panel) and femoral plaque volume (lower panel) in the study population (n = 184). Spearman correlation method was used. **c** BMP12 concentration determined by ELISA in serum from patients (n = 184) with different sized carotid (upper panel; No plaque = 104, < 0.20 = 70, ≥ 0.20 = 10) and femoral plaques (lower panel; No plaque = 84, < 0.20 = 5, ≥ 0.20 = 35). Data are expressed as concentration of BMP12 (pg/mL), presented as mean ± SD and compared using Kruskal–Wallis test. **d** Receiver operating characteristic (ROC) curves of BMP12 to identify patients with femoral plaque presence and femoral plaques above 0.2 mL. **e** ROC curves of the probabilities obtained in multivariate regression analysis to identify femoral plaque presence and femoral plaques above 0.2 mL. AUROC, area under the receiver operating characteristic

Next, we evaluated the relationship between the circulating content of BMP12 and widely known markers of atherosclerosis such as brachial endovascular reactivity and IMT, but no association was found (Fig. S3). Additionally, serum BMP12 levels were evaluated in relation to plaque burden, taking into account both the presence and volumetric extent of lesions in the carotid and femoral arteries, revealing an increased concentration of BMP12 both in patients with carotid plaques (311.86 ± 255.13 vs. 410.99 ± 359.31 pg/mL, p = 0.0464) and in patients with femoral plaques (277.70 ± 232.19 vs. 419.86 ± 347.44 pg/mL, p = 0.0023) (Fig. 5a). Regarding plaque volume, we found significant correlations between circulating BMP12 levels and carotid (r = 0.178, p = 0.016) or femoral (r = 0.287, p < 0.001) plaque volume (Fig. 5b). In order to gain a deeper understanding of the association between serum BMP12 and atherosclerotic plaque volume, we categorized patients according to mean carotid or femoral plaque size. Classification of patients according to mean carotid plaque size did not show any difference in serum BMP12 levels across groups; however, BMP12 levels increased linearly with femoral plaque size, with these differences being statistically significant across the groups (Fig. 5c).

### Serum content of BMP12 is an independent predictor of femoral atherosclerosis

To determine the factors associated with serum BMP12, we performed bivariate analyses of different clinical variables and confounding factors and their association with carotid and femoral plaque volumes. Correlations are shown in Table S1. Remarkably, none of the clinical variables associated with serum BMP12 were significantly associated with either carotid or femoral plaque volume in this cohort. While the association of plaque volume (carotid or femoral) depended on factors such as age, lactate dehydrogenase or glomerular filtration rate, serum BMP12 was associated with HDL cholesterol, triglycerides, gamma-glutamyltransferase (GGT), alkaline phosphatase, and C-reactive protein. We next built multivariate logistic regression models to test the ability of serum BMP12 to serve as an atherosclerosis biomarker. This analysis revealed that beyond traditional and well-understood risk factors for atherosclerosis, such as age, male sex or current smoking status, BMP12 was an independent predictor of atherosclerotic femoral plaque presence (OR = 1.18 for each 100 pg/mL, p = 0.009) (Table 2).

**Table 2:** Univariate and multivariate logistic regression analyses of the independent variables associated with the presence of femoral plaques in the study cohort (n = 184)

Independent variables	Univariate analysis			Multivariate analysis		
	**OR**	**95% CI**	***p* value**	**OR**	**95% CI**	***p*** **value**
Age (years)	1.08	[1.04–1.12]	< 0.001	1.08	[1.04–1.13]	** < 0.001**
Sex (male/female)	2.45	[1.34–4.47]	0.004	3.42	[1.71–6.82]	** < 0.001**
BMI (kg/m^2^)	0.98	[0.93–1.03]	0.505			
Diabetes (yes/no)	2.08	[0.89–4.86]	0.090			
Hypertension (yes/no)	1.50	[0.84–2.70]	0.171			
Previous history of CVD (yes/no)	3.95	[1.41–11.04]	0.009			
Current smoking (yes/no)	1.50	[0.76–2.95]	0.242			
Glycated haemoglobin (%)	1.25	[0.80–1.96]	0.328			
HDL cholesterol (mg/dL)	0.99	[0.97–1.01]	0.358			
Triglycerides (mg/dL)	1.00	[0.99–1.00]	0.445			
GGT (IU/L)	1.00	[0.99–1.01]	0.706			
ALP (IU/L)	0.99	[0.97–1.00]	0.121			
LDH (U/L)	1.01	[1.00–1.02]	0.040			
C-reactive protein (mg/L)	1.08	[0.59–1.96]	0.803			
Serum Creatinine (mg/mL)	1.03	[1.01–1.05]	0.001			
Glomerular filtration rate (mL/min/1.73 m^2^)	0.97	[0.95–0.99]	0.005			
BMP12 (each 100 pg/mL)	1.20	[1.06–1.35]	0.003	1.18	[1.04–1.34]	**0.009**

OR, odds ratio; CI, confidence interval; BMI, body mass index; CVD, cardiovascular disease; HDL, high-density lipoprotein; GGT, gamma-glutamyltransferase; ALP, alkaline phosphatase; LDH, lactate dehydrogenase

In contrast, circulating levels of BMP12 did not resist multivariate adjustment to predict carotid atherosclerosis (Table S2). Subsequently, we built a new model to predict the presence of femoral plaques with a volume over 0.2 mL. This analysis revealed the same independent predictors, although BMP12 lost statistical significance. However, it was kept as a trend in the model since its removal produced significative differences (Table S3). Moreover, we performed receiver operating characteristic (ROC) curve analysis to evaluate the accuracy of circulating BMP12 levels in the diagnosis of femoral atherosclerosis. We tested the presence of plaque, as well as plaques ≥ 0.2 mL, as outcomes for the curves. On the one hand, serum BMP12 alone showed mild to moderate accuracy (area under the ROC curve (AUROC) = 0.630 and 0.703) in detecting the presence of femoral plaques and plaques ≥ 0.2 mL, respectively (Fig. 5d). On the other hand, BMP12 showed no capacity to detect carotid atherosclerosis in the study cohort, regardless of plaque size (Fig. S4). When multivariate models developed to predict femoral plaques were used to build the curves, including age, sex and BMP12, the AUROC values increased to 0.757 for predicting plaque presence (model shown in Table 2) and 0.796 for predicting plaque volume over 0.2 mL (model shown in Table S3) (Fig. 5e).

## Discussion

Vascular calcification is linked to an increased risk of heart disease, stroke, and atherosclerotic plaque rupture [32]. Calcification of the arterial wall, an active and tightly regulated process principally driven by VSMCs, reduces elasticity, resulting in arterial stiffness and constituting an independent predictor of CV risk [33]. In line with previous studies that have demonstrated that BMP2 promotes VSMC calcification by activating genes that facilitate the transition of VSMCs to a synthetic phenotype [34] and enhances VSMC migration through the upregulation of myosin protein [23], this work demonstrates for the first time that BMP12 protein induces both processes in VSMCs, without altering cell proliferation. Indeed, this BMP protein promotes the expression of osteogenic markers (RUNX2 and ALP) and genes related to cellular migration (*Mmp9*), while reducing the contractile marker *Desmin*, whose loss indicates a shift toward a synthetic phenotype [35]. Mechanistically, our data suggest that the pro-atherogenic effects of BMP12 are driven through canonical BMP signaling, since they were blocked using small-molecule inhibitors of type I BMP receptors. These findings support previous data showing that the inhibitor LDN-193189 decreased BMP2-induced vascular calcification [22] and atherosclerosis progression in an atherogenic mouse model [21].

Building on these mechanistic insights, we next investigated whether BMP12 is associated with vascular disease in humans. There are several reports on BMP signaling and how it impacts the mechanisms linked to pathological wall remodeling. In fact, the human transcriptomic data analysis confirmed that not only BMP2 is locally expressed within the human atherosclerotic niche, but also BMP12 was detected in both carotid and femoral plaque tissues, supporting its potential contribution to vascular remodeling. To our knowledge, this is the first study which describes an association between serum BMP12 levels and CVD. Similar to a previous report demonstrating that plasma levels of BMP2 are significantly higher in patients with CAD or T2D [26], we found increased BMP12 levels in the serum of patients with a previous history of CVD and in patients with T2D. In this regard, a previous study of genome-wide profiling and competing endogenous RNA (ceRNA) network analysis suggested increased BMP12 expression in patients with atrial fibrillation compared to healthy controls [36].

In this regard, BMP12 was associated with the LAP and VAI scores, which have been previously related to CVD incidence [12, 13]. While LAP and VAI are superior to standard BMI in reflecting the deleterious endocrine activity of visceral fat, they remain surrogate predictors of CV risk. In contrast, the association of BMP12 with atherosclerosis introduces a direct biological layer, as BMPs are implicated in the molecular pathogenesis of vascular remodeling. Consequently, alterations in BMP12 levels may precede macrovascular anatomical changes, potentially offering an earlier window for clinical intervention than traditional markers. A major advantage of our approach is demonstrated in the multivariate regression models. While LAP and VAI are strongly dependent on metabolic and lipid parameters (such as triglycerides, HDL cholesterol, and waist circumference), our predictive model effectively identifies plaque presence using only BMP12 adjusted for fundamental demographic and lifestyle risk factors (sex, age, and current smoking status), highlighting that BMP12 provides robust, independent predictive value for vascular damage without requiring complex or confounding metabolic variables.

We also describe herein higher BMP12 serum levels in patients with carotid and/or femoral atherosclerosis compared to those without plaques. On the one hand, the increase in patients with carotid atherosclerosis may be related to an increased incidence of stroke. On the other hand, serum BMP12 correlated with both carotid and femoral plaque volume. In line with our results, Zhang et al*.* also described that plasma BMP2 levels correlated positively with plaque volumes [26]. The global prevalence of PAD represents 25.6% of the global burden of CVD [37], and direct healthcare expenditures related to PAD exceed those of CAD, largely driven by a higher burden of polyvascular involvement, frequent CV complications, and elevated annual rates of hospitalization [38]. Furthermore, mortality associated with PAD has risen in recent years, with this increase being much greater in women and the epidemiology expanding to younger populations [39]. Therefore, it is of paramount importance to detect those patients with femoral plaques and thus prevent the development of PAD, which could improve patient quality of life and significantly reduce its economic impact. Although ABI measurements were not available to estimate PAD prevalence, and no association was observed between serum BMP12 levels and other surrogate markers such as IMT or brachial endothelial reactivity in this study cohort, the marked elevation of circulating BMP12 in patients with femoral atherosclerosis suggests a potential link with PAD development.

When we analyzed the potential confounders that might be modifying BMP12 concentrations, we found expected associations between several clinical variables and the presence of carotid or femoral atherosclerosis, such as advanced age and decreased glomerular filtration rate among patients with plaque. Moreover, BMP12 levels appeared to be related to HDL cholesterol, triglycerides, GGT, alkaline phosphatase, and C-reactive protein. Although most of these variables are often altered in patients with CVD, it is important to highlight that none of them were associated with carotid or femoral atherosclerosis in this study cohort, suggesting that the association of BMP12 with atherosclerosis is independent of other clinical variables. To confirm this association, we evaluated all the variables that were associated either with plaque presence or with BMP12 concentrations by performing a logistic regression analysis. We also considered traditional risk factors that could act as potential confounders, such as a previous history of CVD, T2D, AHT, or current smoking status, finding that beyond age and male sex, serum BMP12 content is an independent predictor of femoral atherosclerotic plaque presence (OR = 1.18 for each 100 pg/mL, *p* = 0.009). Indeed, the AUROC for BMP12 increased from 0.757 to 0.796 when stratified by femoral plaque size category. Although these findings suggest that BMP12 may play an important role in atherosclerotic progression and plaque calcification, further research involving larger and more diverse cohorts of individuals with CVD will be essential to validate and extend these results.

Despite the clinical relevance of these findings, several limitations must be acknowledged. First, the sample size and the single-center design of the study may restrict the external validity of our findings and limit their generalizability to broader populations, particularly individuals with different demographic or metabolic profiles, including leaner cohorts. A further limitation is the unavailability of specific data regarding certain pharmacological therapies, such as statins, SGLT2 inhibitors, and GLP-1 receptor agonists, although the analysis accounted for other key treatments such as angiotensin-converting enzyme inhibitors, angiotensin receptor blockers, and oral antidiabetic agents. Furthermore, while BMP12 was used in vitro at 20–50 ng/mL based on established protocols for BMP2 in VSMCs [23, 24], these concentrations are substantially higher than levels detected in systemic circulation. Although this discrepancy is likely explained by local microenvironmental accumulation or extracellular matrix sequestration, future studies directly quantifying local tissue concentrations of BMP12 within human atherosclerotic plaques are required to confirm this hypothesis.

Taken together, the findings presented in this study provide the first evidence that BMP12 is involved in pathological vascular wall remodeling, promoting VSMC migration and calcification through canonical BMP signaling. Moreover, serum levels of BMP12 are increased in CVD, positioning this BMP protein as an independent predictor of femoral atherosclerosis. Hence, pending validation in larger cohorts, we propose BMP12 as a novel biomarker of femoral atherosclerosis with potential clinical applications in PAD prevention.

## Materials and methods

### Cell culture

Animal procedures were carried out in accordance with Spanish legislation and guidelines from Directive 2010/63/EU of the European Parliament on the protection of animals used for scientific purposes. Animals were housed under controlled conditions of temperature (22 °C) and humidity with dark/light cycles of 12 h at the Universidad Autónoma de Madrid (Madrid, Spain) animal facilities. Mice were fed standard chow diet *ad libitum* and had free access to drinking water. Primary VSMCs were isolated from the thoracic aorta of male C57BL6J mice (n = 4, 10–12 weeks) following the explant technique. Mice were anesthetized using intraperitoneal injection of ketamine (125 mg/kg, Pfizer) and xylazine hydrochloride (25 mg/kg, Bayer). The depth of anesthesia was confirmed by the absence of pedal and corneal reflexes. Once deep anesthesia was achieved, euthanasia was performed by terminal exsanguination via cardiac puncture. The thoracic cavity was then opened, and the aorta was carefully isolated and placed in ice-cold PBS supplemented with 1% penicillin–streptomycin for further processing. Under sterile conditions, the explants obtained were incubated in high glucose Dulbecco’s Modified Eagle medium (DMEM, Cytiva), supplemented with collagenase (C6885, Merck Life Science) and antibiotics (penicillin/streptomycin) for 1 h at 37 °C and 5% CO_2_. Aortas were then washed and cultured in DMEM supplemented with HEPES, antibiotics and 10% heat-inactivated fetal bovine serum (FBS).

The immortalized VSMC cell line was kindly provided by Dr. Almudena Gómez Hernández (Universidad Complutense de Madrid). These cells were obtained by the immortalization of primary mouse VSMCs using SV40 large antigen. Cells were cultured in DMEM supplemented with HEPES, antibiotics, and 10% FBS.

All cells were cultured at 37 °C with 5% CO_2_ and a relative humidity of 95%.

### Pharmacological inhibition of type I BMP receptors

The pharmacological inhibitors of type I BMP receptors used in this study were LDN-193189 and DMH2 (SML-0559, Merck Life Science; 5580, Tocris Bioscience, respectively). The inhibitors’ affinity for each receptor and their working concentrations are shown in Table S4. The inhibitors were added as a pre-treatment 1 h prior to BMP stimulation in VSMCs.

### Cell viability and proliferation assessment

For the proliferation assessment, VSMCs were cultured (60% confluence) in DMEM 0% FBS and were incubated in either DMEM with 0% or 10% FBS, in the presence or absence of recombinant mouse BMP12 protein (20 or 50 ng/mL, 779-G7, R&D Systems) for 24 or 48 h. For the cell viability assay, VSMCs were cultured in DMEM 0% FBS for 24 h, in the absence or presence of the effective doses of type I BMP receptors inhibitors (Fig. S5).

Both assays were evaluated via crystal violet staining. After washing away non-adherent/dead cells with PBS, plates were incubated for 30 min at room temperature with 0.2% crystal violet (212,120,250, Thermo Fisher Scientific Inc.) diluted in 2% ethanol. Excess dye was removed with distilled water (dH_2_O) and left to dry; the bound stain was dissolved in 1% sodium dodecyl sulfate (SDS). Absorbance was then recorded at 560 nm using a Spectra MR spectrophotometer (29,010, Dynex Technologies), in which absorbance is directly proportional to the number of viable cells.

### Calcification assay

VSMCs play a key role in the pathogenesis of vascular calcification, and one of the stimuli involved in the osteogenic differentiation of these cells is hyperphosphatemia [40, 41]. To induce phosphate (Pi)-mediated calcification, cells were incubated with calcifying medium (CalM: DMEM 0% FBS plus 3.5 mM Na_2_HPO_4_, pH 7.4). CalM was used in the presence or absence of recombinant BMP12 protein (20 ng/mL). For primary VSMCs, medium was changed every 3 days for a total of 10 days of incubation. In the case of immortalized VSMCs, cells were collected after 3 days of calcification induction. Then, cells were washed twice with PBS without Ca^2+^ and Mg^2+^, and fixed with 4% paraformaldehyde (PFA) for 15 min at room temperature. Subsequently, cells were washed with dH_2_O and stained with Alizarin Red S (40 mM, pH 4.2) (A5533, Merck Life Science) for 30 min. Then, the staining was removed, and cells were washed with dH_2_O. Extracellular Ca^2+^ deposition was stained red. Representative images were taken using a Nikon D5100 camera coupled to an inverted microscope (Leica).

For staining quantification, VSMCs were incubated in the presence of 10% acetic acid for 30 min with shaking. Cells were collected using a cell scraper into a centrifuge tube, vortexed, heated at 85 °C for 10 min, and then cooled on ice for 5 min. After centrifugation at 13,000 rpm for 15 min, the supernatant was transferred to a new tube and mixed with 3% NH_4_OH to a final pH ranging between 4.1 and 4.5. The absorbance of each sample was measured with a Spectra MR spectrophotometer (29,010, Dynex Technologies) at 405 nm. Samples were normalized using a standard curve.

### Wound healing assay

Migration was assessed by the wound healing assay, as described by Liang et al. [42]. VSMCs were cultured in DMEM 0% FBS. Cells were scratched using a sterile yellow tip and washed with PBS to remove cell debris. Then, VSMCs were stimulated with vehicle or recombinant mouse BMP12 protein (20 ng/mL, 779-G7, R&D Systems) for 24 h. Representative phase-contrast images (4X) of the wound were taken at 0 and 24 h using a Nikon D5100 camera coupled to an inverted microscope (Leica). Cell migration was quantified by measuring the area of the wound (A) using ImageJ software (NIH), and the relative migration percentage was calculated as follows:

$$relative\ migration(\%)=\frac{A0 h-A24 h}{A0h}\times 100$$

### Migration assay by Matrigel

Extracellular matrix-dependent remodeling behavior was evaluated using a Matrigel-based segment formation assay as previously described [43], with minor modifications. First, 130 μL of Matrigel matrix (Corning® Matrigel® Growth Factor Reduced (GFR) Basement Membrane Matrix) was added under sterile conditions to TC-treated 48-well plates (Corning® Costar®) and was polymerized for 30 min at 37 °C. Then, immortalized VSMCs were cultured in the Matrigel matrix at a concentration of 10^5^ cells/mL in DMEM 0% FBS. Cells were cultured for 6 h at 37 °C in a constant atmosphere of 5% CO_2_ and relative humidity of 95%. Representative phase-contrast images of the segment formation were taken at 6 h using a Nikon D5100 camera coupled to an inverted microscope (Leica). Cell migration was quantified by measuring the segment formation using the Angiogenesis plugin for ImageJ software (NIH).

### Gene expression analysis

Total RNA isolation was carried out using TRIzol reagent (Vitro, Sevilla, Spain)//TRItidy G (A4051, AppliChem GmbH). RNA concentration and purity were subsequently determined via a NanoDrop 2000c spectrophotometer (Thermo Fisher Scientific, Inc.). Reverse transcription of the extracted RNA was performed using the ImProm-II™ Reverse Transcription System (Promega Inc.) in a T100™ Thermal Cycler (Bio-Rad Laboratories, Inc.) to synthesize cDNA. Quantitative real-time PCR (RT-qPCR) was then conducted via the SYBR Green detection method on a StepOnePlus™ Real-Time PCR System (Thermo Fisher Scientific, Inc.), with relative quantification calculated using the ΔΔCt method. All samples were analyzed in duplicate and normalized against the *36b4* house-keeping gene. Specific primer sequences are provided in Table S5.

### Protein analysis by Western blot

Total protein was harvested from VSMCs by scraping cells in RIPA lysis buffer (50 mM Tris–HCl, pH 7.4, 1% Triton X-100, 0.2% SDS, 1 mM EDTA, 1 mM PMSF, and 5 μg/mL leupeptin). Protein lysates were denatured by boiling in Laemmli sample buffer and subsequently resolved on 10% SDS-PAGE gels. Following electrophoretic separation, proteins were transferred onto nitrocellulose membranes (Bio-Rad). Blocked membranes were then incubated overnight at 4 °C with the following primary antibodies: anti-Vinculin (sc-73614, Santa Cruz Biotechnology Inc.), anti-RUNX2 (ab192256, Abcam), anti-ALP (ab229126, Abcam), anti-ALK2 (sc-374523, Santa Cruz Biotechnology Inc.), anti-ALK3 (ABD51, Sigma-Aldrich), anti-ALK6 (sc-515886,Santa Cruz Biotechnology Inc.), anti-phospho-SMAD1 (Ser463/465)/SMAD5 (Ser463/465)/SMAD9 (Ser 465/469) (#13,820, Cell Signalling Technology) and anti-Smad1 (#9743, Cell Signaling Technology). Afterwards, membranes were incubated with the corresponding secondary antibodies: anti-rabbit (sc-2357, Santa Cruz Biotechnology Inc.) or anti-mouse (#32,430, Thermo Fisher Scientific). Immunoreactive bands were visualized using the ECL Western blotting protocol (Bio-Rad). Densitometric analysis of the bands was performed using ImageJ software (NIH).

### Analysis of BMP human transcriptomic data

Gene expression levels of *BMP2* and *BMP12* were obtained from the National Center for Biotechnology Information Gene Expression Omnibus (GEO) database following a search and selection of transcriptomic studies involving human atherosclerotic plaques. Two independent datasets were selected for analysis: GSE100927 [44], and GSE120521 [45].

### Study population

For this cross-sectional clinical cohort, patients were recruited from Hospital Universitario La Princesa (Madrid, Spain) over a 6-month period. Exclusion criteria included daily alcohol consumption exceeding 20 g, a history of asthma or malignancy, and the presence of any severe concomitant medical condition. Individuals were likewise excluded if laboratory testing revealed iron overload, positivity for autoantibodies, or active infections with hepatitis B virus, hepatitis C virus, or human immunodeficiency virus. Finally, individuals taking active drugs, including cannabis and cocaine, were excluded from the study.

### Demographic, clinical and biochemical assessment

All study participants underwent a comprehensive clinical evaluation, which included a detailed interview focused specifically on smoking habits, alcohol consumption, substance use, medication use, and a history of T2D and arterial hypertension, as well as measurements of weight and height. Venous blood samples from each participant were obtained after an overnight fast in order to test serum levels of different biochemical and metabolic parameters. Insulin resistance was calculated using the homeostatic model assessment of insulin resistance (HOMA IR) [46]. The LAP and VAI scores were calculated as previously described [47, 48].

### Assessment of vascular damage

Brachial endovascular reactivity was evaluated using flow-mediated dilation. Briefly, the difference in diameter suffered by the right brachial artery at rest and after subjecting the arm to ischemia for 4 min was measured and expressed as a percentage, as previously described [49]. For IMT assessments, the methodological framework defined in the Mannheim consensus [50] was followed. Each subject underwent three measurements per side, which were recorded and quality-assessed by two experienced vascular radiologists (AF, JMM). The mean value of all readings meeting predefined quality benchmarks was used to determine carotid or femoral IMT. Evaluation of arterial plaque volume was performed in each patient via ultrasonography (Aplio XG, Canon) according to internationally accepted criteria [51]. A lesion was defined as an arterial plaque if it satisfied at least two of the following conditions: (1) an intima-media thickness > 1.5 mm, (2) an impression in the vascular lumen, or (3) an abnormal wall texture. Plaque burden was evaluated in both carotid arteries (encompassing 2 cm of the distal common carotid and 1 cm of the distal internal carotid) and femoral arteries (encompassing the distal 2 cm of common femoral arteries and 1 cm proximal to their bifurcation). Total plaque burden was represented as the cumulative volume of all detected plaques. All sonographic acquisitions were carried out by two expert vascular radiologists (AF, JMM) using a 7 MHz linear probe (PLT-704SB, Canon) and a high-frequency volumetric linear probe (PLT-1204 MV, Canon) coupled with 3D/4D volumetric reconstruction software (Toshiba UIMV-A500A, Canon).

### Quantitative analysis of serum BMP12 levels

Serum levels of BMP12 were determined by ELISA using the human GDF7 ELISA kit (MBS2505805, MyBioSource, Inc.), in accordance with the manufacturer’s instructions. Sample absorbances were measured against a standard curve fitted with a four-parameter logistic (4-PL) model. This assay provides a limit of detection (LOD) of 37.5 pg/mL and a functional detection range (LOQ) of 62.5–4000 pg/mL. The assay demonstrated high precision (intra- and inter-assay CV < 10%) and accuracy, with spike-recovery rates of 96%–110% and dilutional parallelism of 89%–107%. No significant cross-reactivity with structural analogs was observed. To ensure analyte stability, blood samples were processed within 1 h of collection, and serum aliquots were stored at −80 °C. All samples were measured in duplicate, and repeated freeze–thaw cycles were strictly avoided.

### Statistical analysis

The normality of data distribution was assessed using the Kolmogorov–Smirnov test. Categorical variables are reported as absolute numbers (n) and relative percentages (%). Continuous data are summarized as mean ± standard deviation (SD) for clinical parameters or mean ± standard error of the mean (SEM) for experimental measurements. Inter-group comparisons for qualitative variables were performed using Pearson's χ2 test or Fisher's exact test, as appropriate. Bivariate correlations between quantitative variables were calculated using Spearman's rho (ρ) correlation. For continuous variables with a normal distribution, Student's t-test was applied for independent group comparisons, whereas the Mann–Whitney U test was utilized for non-normally distributed data. Differences across multiple group comparisons were evaluated using one-way and two-way analysis of variance (ANOVA). To determine factors associated with the presence of carotid and femoral atherosclerotic plaques, logistic regression models were constructed with adjustment for potential confounding variables. Univariate and multivariate analyses were performed, with predictor selection based on the likelihood ratio test. The assumption of linearity of the logit was formally assessed using the Box-Tidwell procedure. The goodness of fit was evaluated using the Hosmer–Lemeshow statistic. All statistical analyses were performed using GraphPad Prism 6.0 (GraphPad Software Inc.) and IBM SPSS Statistics 26.0 (SPSS Inc., IBM) software, with a p-value < 0.05 considered statistically significant.

## Supplementary Information

Supplementary Material 1.

## Data Availability

All data supporting the findings of this study are available within the paper and its Supplementary Information files. The public transcriptomic datasets analyzed in this study are available in the NCBI Gene Expression Omnibus (GEO) database under accession number GSE100927 and GSE120521. The raw datasets generated and analyzed during the current study are not openly available due to privacy and ethical restrictions but are available from the corresponding author upon reasonable request.
