# Supplementary material for: Bone morphogenetic protein 12: a novel target for cardiovascular disease
Source: Mol Biomed. 2026 Oct 29;7:232. doi: 10.1186/s43556-026-00609-y (PMC13719908; doi:10.1186/s43556-026-00609-y)
Supplement: Supplementary file 1 [file 43556_2026_609_MOESM1_ESM.docx]

## **TITLE: Bone morphogenetic protein 12: a novel target for cardiovascular disease**

**AUTHORS:** Stephania C. Isaza^1^, Miguel Á. Hernández-García^1^, Carlos Ernesto Fernández-García^1*^, Carolina Pérez-Utrera^2^, María Álvarez-Rubal^1^, José M. Muñoz^3^, Alfonsa Friera^3^, Carmelo García-Monzón^1^, Almudena Gómez-Hernández^4^, Pedro Landete^5,6,9^, Javier Egea^1^, Fernando Alfonso^6,7,8^, Águeda González-Rodríguez^2,9*^

**AFFILIATIONS:**

^1^Unidad de Investigación, Hospital Universitario Santa Cristina, Instituto de Investigación Sanitaria-Princesa IIS-IP, Madrid, Spain.

^2^Instituto de Investigaciones Biomédicas Sols-Morreale (IIBM), Consejo Superior de Investigaciones Científicas-Universidad Autónoma de Madrid, Spain.

^3^Servicio de Radiodiagnóstico, Hospital Universitario de La Princesa, Instituto de Investigación Sanitaria-Princesa IIS-IP, Madrid, Spain.

^4^Laboratorio de Enfermedades Hepáticas y Vasculares, Departamento de Bioquímica y Biología Molecular, Facultad de Farmacia, Universidad Complutense de Madrid, Madrid, Spain.

^5^Servicio de Neumología, Hospital Universitario de La Princesa, Instituto de Investigación Sanitaria-Princesa IIS-IP, Madrid, Spain.

^6^Universidad Autónoma de Madrid, Madrid, Spain.

^7^Servicio de Cardiología, Hospital Universitario de La Princesa, Instituto de Investigación Sanitaria-Princesa IIS-IP, Madrid, Spain.

^8^Centro de Investigación Biomédica en Red de Enfermedades Cardiovasculares (CIBERCV), Madrid, Spain.

^9^Centro de Investigación Biomédica en Red de Diabetes y Enfermedades Metabólicas Asociadas (CIBERDEM), Madrid, Spain.

***Correspondence**: Águeda González-Rodríguez and Carlos Ernesto Fernández-García

Email: [aguedagr@](mailto:aguedagr@)iib.uam.es; cernesto.fernandez.garcia@gmail.com.

**Supplementary Table 1. Correlations between clinical variables, atherosclerotic plaque volume and serum BMP12 (n=184).**

| **Independent variables** | **Carotid plaque mean volume** | | **Femoral plaque mean volume** | | **Serum BMP12** | |
| --- | --- | --- | --- | --- | --- | --- |
|  | **r** | ***p* value** | **r** | ***p* value** | **r** | ***p* value** |
| Age (years) | **0.428** | **<0.001** | **0.374** | **<0.001** | 0.142 | 0.054 |
| Body mass index (kg/m^2^) | **0.196** | **0.008** | -0.011 | 0.881 | 0.102 | 0.169 |
| Glucose (mg/dL) | 0.069 | 0.353 | 0.093 | 0.210 | 0.041 | 0.580 |
| Glycated haemoglobin (%) | 0.128 | 0.082 | **0.174** | **0.018** | 0.134 | 0.070 |
| HOMA-IR (a.u.) | 0.073 | 0.323 | 0.048 | 0.520 | 0.032 | 0.664 |
| Total cholesterol (mg/dL) | -0.088 | 0.237 | -0.094 | 0.206 | -0.059 | 0.427 |
| LDL cholesterol (mg/dL) | **-0.176** | **0.017** | -0.075 | 0.311 | -0.048 | 0.514 |
| HDL cholesterol (mg/dL) | 0.037 | 0.614 | -0.091 | 0.217 | **-0.155** | **0.036** |
| Triglycerides (mg/dL) | <0.001 | 0.999 | 0.026 | 0.722 | **0.179** | **0.015** |
| ALT (IU/L) | 0.013 | 0.859 | 0.034 | 0.651 | 0.107 | 0.150 |
| AST (IU/L) | 0.038 | 0.613 | 0.116 | 0.116 | 0.079 | 0.289 |
| GGT (IU/L) | -0.026 | 0.723 | 0.047 | 0.530 | **0.243** | **0.001** |
| Alkaline phosphatase (IU/L) | -0.031 | 0.680 | -0.075 | 0.309 | **0.177** | **0.016** |
| Lactate dehydrogenase (IU/L) | **0.189** | **0.011** | **0.153** | **0.041** | 0.134 | 0.073 |
| C reactive protein (mg/L) | 0.080 | 0.278 | 0.127 | 0.087 | **0.285** | **<0.001** |
| Albumin (g/dL) | **-0.288** | **<0.001** | -0.033 | 0.652 | -0.111 | 0.133 |
| Platelets (10^9^/L) | **-0.151** | **0.041** | -0.113 | 0.127 | 0.088 | 0.233 |
| Serum creatinine (mg/dL) | 0.112 | 0.131 | **0.194** | **0.008** | 0.035 | 0.640 |
| Glomerular filtration rate (mL/min per 1.73m^2^) | **-0.222** | **0.003** | **-0.178** | **0.015** | -0.039 | 0.596 |
| Serum BMP12 (pg/mL) | **0.177** | **0.016** | **0.288** | **<0.001** | - | - |

Data are shown as mean ± standard deviation or as number of cases (%). HOMA-IR, homeostatic model assessment-insulin resistance; HDL, high-density lipoprotein; LDL, low-density lipoprotein; ALT, alanine aminotransferase; AST, aspartate aminotransferase; GGT, gamma-glutamyltransferase.

**Supplementary Table 2. Univariate and multivariate logistic regression analysis of the independent variables associated with presence of carotid plaques (n=184).**

| **Independent variables** | **Univariate analysis** | | | **Multivariate analysis** | | |
| --- | --- | --- | --- | --- | --- | --- |
|  | **OR** | **95% CI** | ***p* value** | **OR** | **95% CI** | ***p* value** |
| Age (years) | 1.10 | [1.06-1.15] | <0.001 | 1.12 | [1.06-1.17] | **<0.001** |
| Sex (male/female) | 0.88 | [0.49-1.59] | 0.674 |  |  |  |
| BMI (kg/m^2^) | 1.07 | [1.01-1.13] | 0.017 |  |  |  |
| Diabetes (yes/no) | 1.27 | [0.57-2.78] | 0.571 |  |  |  |
| Hypertension (yes/no) | 3.03 | [1.67-5.56] | <0.001 |  |  |  |
| Previous history of CVD (yes/no) | 6.67 | [2.38-20.00] | <0.001 | 5.26 | [1.67-16.67] | **0.005** |
| Current smoking (yes/no) | 2.13 | [1.09-4.17] | 0.027 | 5.26 | [2.50-12.50] | **<0.001** |
| LDL cholesterol (mg/dL) | 0.99 | [0.98-1.00] | 0.020 |  |  |  |
| HDL cholesterol (mg/dL) | 1.01 | [0.99-1.02] | 0.473 |  |  |  |
| Triglycerides (mg/dL) | 1.00 | [0.99-1.00] | 0.458 |  |  |  |
| GGT (IU/L) | 1.00 | [0.99-1.01] | 0.977 |  |  |  |
| ALP (IU/L) | 0.99 | [0.98-1.01] | 0.313 |  |  |  |
| LDH (U/L) | 1.01 | [1.00-1.02] | 0.009 |  |  |  |
| C reactive protein (mg/L) | 0.94 | [0.51-1.71] | 0.832 |  |  |  |
| Albumin (g/dL) | 0.05 | [0.01-0.19] | <0.001 | 0.03 | [0.01-0.16] | **<0.001** |
| Platelets (10^9^/L) | 0.01 | [0.00-1.42] | 0.066 |  |  |  |
| Glomerular filtration rate (mL/min/1.73 m^2^) | 0.98 | [0.96-0.99] | 0.012 |  |  |  |
| BMP12 (each 100pg/mL) | 1.11 | [1.01-1.23] | 0.035 |  |  |  |

OR, odds ratio; CI, confidence interval; BMI, body mass index; CVD, cardiovascular disease; LDL, low-density lipoprotein; HDL, high-density lipoprotein; GGT, gamma-glutamyltransferase; ALP, alkaline phosphatase; LDH, lactate dehydrogenase.

**Supplementary Table 3. Univariate and multivariate logistic regression analysis of the independent variables associated with presence of ≥0.2 mL femoral plaques (n=184).**

| **Independent variables** | **Univariate analysis** | | | **Multivariate analysis** | | |
| --- | --- | --- | --- | --- | --- | --- |
|  | **OR** | **95% CI** | ***p* value** | **OR** | **95% CI** | ***p* value** |
| Age (years) | 1.07 | [1.03-1.13] | 0.002 | 1.11 | [1.05-1.17] | **<0.001** |
| Sex (male/female) | 3.36 | [1.38-8.17] | 0.008 | 5.58 | [2.03-15.32] | **0.001** |
| BMI (kg/m^2^) | 0.96 | [0.90-1.04] | 0.330 |  |  |  |
| Diabetes (yes/no) | 1.81 | [0.72-4.50] | 0.205 |  |  |  |
| Hypertension (yes/no) | 2.41 | [1.12-5.20] | 0.025 |  |  |  |
| Previous history of CVD (yes/no) | 3.57 | [1.44-8.85] | 0.006 |  |  |  |
| Current smoking (yes/no) | 2.34 | [1.07-5.11] | 0.032 | 4.34 | [1.61-11.70] | **0.004** |
| Glycated haemoglobin (%) | 1.25 | [0.78-2.01] | 0.342 |  |  |  |
| HDL cholesterol (mg/dL) | 0.99 | [0.97-1.02] | 0.601 |  |  |  |
| Triglycerides (mg/dL) | 1.00 | [1.00-1.00] | 0.901 |  |  |  |
| GGT (IU/L) | 1.01 | [1.00-1.02] | 0.171 |  |  |  |
| ALP (IU/L) | 1.00 | [0.98-1.02] | 0.917 |  |  |  |
| LDH (U/L) | 1.00 | [0.99-1.01] | 0.810 |  |  |  |
| C reactive protein (mg/L) | 1.10 | [0.53-2.26] | 0.804 |  |  |  |
| Serum Creatinine (µg/mL) | 1.01 | [0.99-1.03] | 0.577 |  |  |  |
| Glomerular filtration rate (mL/min/1.73 m^2^) | 1.00 | [0.97-1.02] | 0.775 |  |  |  |
| BMP12 (each 100pg/mL) | 1.17 | [1.05-1.31] | 0.004 | 1.11 | [0.98-1.26] | **0.090** |

OR, odds ratio; CI, confidence interval; BMI, body mass index; CVD, cardiovascular disease; HDL, high-density lipoprotein; GGT, gamma-glutamyltransferase; ALP, alkaline phosphatase; LDH, lactate dehydrogenase.

**Supplementary Table 4. Pharmacological inhibitors of type I BMP receptors.**

|  | **Ki ALK2**  **(nM)** | **Ki ALK3**  **(nM)** | **Ki ALK6**  **(nM)** | **Working concentration** | **Control** |
| --- | --- | --- | --- | --- | --- |
| **LDN-193189** | 5 | 30 | - | 300 nM | - |
| **DMH2** | 43 | 5.4 | <1 | 400 nM | DMSO |

ALK2, activin receptor type-1; ALK3, bone morphogenetic protein receptor type 1A; ALK6, bone morphogenetic protein receptor type 1B; DMSO, dimethyl sulfoxide; Ki, inhibitory constant.

**Supplementary Table 5. Primer sequences for RT-qPCR.**

| **Gene** | **Forward (5’-3’)** | **Reverse (5’-3’)** |
| --- | --- | --- |
| ***m-Acta2*** | CCCAGACATCAGGGAGTAATGG | TCTATCGGATACTTCAGCGTCA |
| **m-*Alk2*** | TGCTAATGATGATGGCTTTCC | CCTTCACAGTGGTCCTCGTT |
| **m-*Alk3*** | ATGCAAGGATTCACCGAAAG | AACAACAGGGGGCAGTGTAG |
| **m-*Alk6*** | CTCCCTCTGCTGGTCCAAAGGACA | CCAGCTGGCTTCCTCCGTGGT |
| ***m-Alpl*** | GGGACGAATCTCAGGGTACA | AGTAACTGGGGTCTCTCTCTTT |
| **m-*Desmin*** | ATGAGACCATCGCGGCTAAG | GGCTGCCTGAGTCAAGTCTGA |
| **m-*Mmp9*** | GCTGACTACGATAAGGACGGCA | TAGTGGTGCAGGCAGAGTAGGA |
| **m-*Opn*** | GACCACCATGGACGACGATG | TGGAACTTGCTTGACTATCGA |
| ***m-Rnx2*** | CCGGTCTCCTTCCAGGAT | GGGAACTGCTGTGGCTTC |
| ***m-36b4*** | AGATGCAGCAGATCCGCAT | GTTCTTGCCATCAGCACC |

*Acta2*, α-smooth muscle actin; *Alk2*, activin receptor type-1; *Alk3*, bone morphogenetic protein receptor type 1A; *Alk6*, bone morphogenetic protein receptor type 1B; *Alpl*, alkaline phosphatase; *Desmin*, desmin; *Mmp9*, matrix metalloproteinase 9; *Opn*, osteopontin; *Rnx2*, runt-related transcription factor 2; *36b4*, ribosomal protein lateral stalk subunit P0.

**Fig. S1 Validation of *BMP2* and *BMP12* mRNA expression levels in public transcriptomic datasets of human atherosclerotic plaques.** Expression levels of *BMP2* and *BMP12* genes from the National Center for Biotechnology Information Gene Expression Omnibus database. **a** *BMP2* and *BMP12* mRNA expression levels obtained from atherosclerotic lesions from carotid (n=29) and femoral (n=26) arteries, derived from the GSE100927 dataset. **b** *BMP12* mRNA expression levels obtained from atherosclerotic lesions from carotid (n=4), derived from the GSE120521 dataset. Data are presented as mean ± SD.

**Fig. S2 BMP12 concentration is not associated with proliferation on VSMCs.** Representative phase-contrast images (4X, scale bar 500 µm) and quantification of proliferation by crystal violet staining at 24h and 48h. Data are expressed as fold of change relative to control condition (10% FBS at 24h), presented as mean ± SEM and compared using one-way ANOVA with Tukey’s post hoc test. Experimental conditions: a murine VSMCs cell line was treated with recombinant mouse BMP12 (20 or 50 ng/mL), in absence or presence of serum (FBS) for 24 or 48h (n=9 for each control condition (C) and n=6 for each BMP12 condition).

**Fig. S3 BMP12 concentration is not associated with markers of atherosclerosis.** BMP12 concentration determined by ELISA in serum from the study population (n=184) according to endovascular reactivity (**a-b**; <7%=60, ≥7%=124), carotid intima media thickness (cIMT, **c-d**; <0.9=82, ≥0.9=12) and femoral intima media thickness (fIMT, **e-f**; <0.9=104, ≥0.9=80). Spearman correlation method was used and data are expressed as concentration of BMP12 (pg/mL), presented as mean ± SD and compared using Mann-Whitney U test.

**Fig. S4 Serum BMP12 diagnostic accuracy in carotid atherosclerosis.** Receiver operating characteristic (ROC) curves of BMP12 to identify patients with carotid plaque presence (a) and carotid plaques above 0.2 mL (b). AUROC, area under the receiver operating characteristic.

**Fig. S5 Combined treatment of VSMCs with the inhibitors LDN or DMH2 and/or BMP12 did not affect cell viability.** Quantification of crystal violet staining. Data are expressed as a percentage of cell viability relative to the control condition and are presented as mean ± SEM. Experimental conditions: VSMCs pretreated for 1 h with the inhibitors LDN-193189 (LDN, 300 nM), DMH2 (400 nM), or vehicle (Control), and subsequently incubated in the presence of BMP12 at 20 or 50 ng/mL for 24 hours (n=6 for each condition).
